# Reliability and validity of OpenPose for measuring HKA angle in dynamic walking videos in patients with knee osteoarthritis

**DOI:** 10.1038/s41598-025-09627-2

**Published:** 2025-07-07

**Authors:** Fanghong Ge, Changjiang Wu, Fangjun Ge, Shenghao Xu, Jianlin Xiao

**Affiliations:** 1 Department of Orthopedics, China-Japan Union Hospital of Jilin University, Changchun, Jilin People’s Republic of China; 2 Zhiyuan Research Institute, Hangzhou, Zhejiang People’s Republic of China; 3 School of Biology and Biological Engineering, South China University of Technology, Guangzhou, Guangdong People’s Republic of China

**Keywords:** OpenPose, Knee osteoarthritis, Hip-knee-ankle angle, Dynamic walking videos, Medical research, Bone

## Abstract

The hip-knee-ankle (HKA) angle is essential to assess surgical evaluation and disease progression in patients with knee osteoarthritis (KOA). Rapid, radiation-free assessment methods are a key area of research. This study investigates the reliability and validity of OpenPose, video-based human pose estimation method, for determining the HKA angle in KOA patients. In this study, we analyzed 50 knees affected by osteoarthritis. The HKA angle was measured using the pose estimation method and X-ray imaging before total knee arthroplasty. The pose estimation method demonstrated excellent test-retest reliability (ICC 1,1 = 1.000) and good consistency with radiography (ICC 2,1 = 0.897), with linear regression analysis showing a good correlation (R^2^ = 0.814). Compared with radiography, the pose estimation method exhibited a fixed error of 0.131°. This is the first study to examine the feasibility of measuring the HKA angle from frontal-view videos of patients walking normally by using the pose estimation method. Using the pose estimation method to measure the HKA angle in knee osteoarthritis patients is reliable and valid. The pose estimation method provides a safe, cost-effective, and user-friendly solution for monitoring lower limb alignment, with promising applications in remote healthcare and rehabilitation management. It eliminates radiation exposure, avoiding the health risks associated with X-ray imaging, and it does not require specialized medical equipment, enabling fully automated analysis.

## Introduction

Knee osteoarthritis (KOA) is increasingly prevalent among the aging population^1^and accurate assessment of lower limb alignment is critical for both monitoring disease progression and guiding treatment such as total knee arthroplasty (TKA)^2^. The hip-knee-ankle (HKA) angle^3^typically measured on coronal plane standing X-rays, is the standard indicator for evaluating lower limb alignment^4,5^. However, X-ray-based methods^6^ have limitations including radiation exposure, high costs, and limited accessibility for routine or at-home use. While static HKA assessment is clinically established, dynamic HKA measurement—capturing alignment during walking—may better reflect the real biomechanical stresses on the knee joint and provide more meaningful insight into functional joint performance and OA progression. Despite its clinical potential, dynamic HKA remains underexplored due to technical challenges in capturing motion data accurately and efficiently.

With advancements in AI and computer vision, markerless pose estimation tools like OpenPose (a video-based human pose estimation method) offer a promising, low-cost, and non-invasive alternative for human motion analysis using simple video recordings^7^. However, while pose estimation has been used in general biomechanics and some post-surgical assessments, its application to dynamic HKA evaluation in KOA patients is virtually unexplored^8^. Existing studies either rely on static images or require clinical staff and specialized setups, limiting real-world usability. This study is the first to investigate the feasibility, reliability, and validity of using OpenPose to measure the HKA angle from frontal-view walking videos of KOA patients. By bridging the gap between clinical needs and emerging technologies, our work contributes a novel, accessible approach for dynamic lower limb alignment assessment, potentially enabling widespread and routine gait monitoring outside clinical settings^9^.

In this study, we aim to evaluate the accuracy of pose estimation software compared to conventional full-length, weight-bearing X-ray imaging in measuring the HKA angle. Specifically, we hypothesize that imageless determination of the HKA angle using OpenPose—a markerless, AI-based motion capture tool—can achieve comparable accuracy to standard radiographic assessments. To test this, we will use OpenPose and conventional X-rays to measure the HKA angle and compare the results obtained from both methods. Our goal is to assess whether 2D video-based gait analysis using widely accessible devices such as smartphones can serve as a reliable and valid tool for clinical and rehabilitation applications. By tracking anatomical landmarks (e.g., hip, knee, ankle) during gait, OpenPose estimates dynamic changes in coronal limb alignment. This study also explores the feasibility of using OpenPose as a safe, affordable, and convenient method to monitor lower limb alignment in patients undergoing TKA.

## Methods

### Study design and patients

We included patients with KOA undergoing TKA between February 2023 and October 2023. The sample was selected from the picture archiving and communication system database at our hospital. The only criterion for exclusion was the inability to stand, which could affect measurement accuracy; yet, there were no cases met this criterion. 50 knees in total were evaluated using preoperative X-rays. All patients submitted written informed consent. This study was approved by the Institutional Review Board of the China-Japan Union Hospital of Jilin University and conducted in accordance with the principles of the Declaration of Helsinki. To determine the sample size, we used GPower 3.1.9.6. For linear regression analysis, 25 participants were required to achieve an effect size (f^2^) > 0.35, with a significance level of 0.05 and a power of 0.80. Therefore, our study analyzed 50 knees. Table 1 summarizes the demographic information for the patients we included.

**Table 1:** Baseline characteristics of KOA patients.

Parameter	No. of patients (*n* = 50)
Age	
> 60	40 (80%)
≤ 60	10 (20%)
Gender	
Male	18 (36%)
Female	32 (64%)
HSS scores	
> 50	31 (62%)
≤ 50	19 (38%)

### Data collection and data analysis

A system was developed using a standard smartphone (XIAOMI REDMI GO F1) with a 600 × 1000 pixel resolution. It has an average frame rate of 30 frames per second. The smartphone was positioned perpendicular to the walkway of the subject, who walked in a straight line 3 m towards the phone. The camera was placed at an appropriate distance to capture the entire body within its field of view. Video data were transferred from the smartphone to a computer over a wireless network and processed using OpenPose.

We measured the HKA angle before TKA using OpenPose and radiography. For OpenPose, a scorer used a digital camera to record each patient’s normal gait. The pose estimation algorithm provided the coordinates of the joint points of hip, knee, and ankle. Then we used Python to process these data. The Euclidean distance between each pair of joint points was calculated to determine the segment lengths, and the cosine theorem was applied to calculate the knee joint angle in each video frame.

**Fig. 1:**
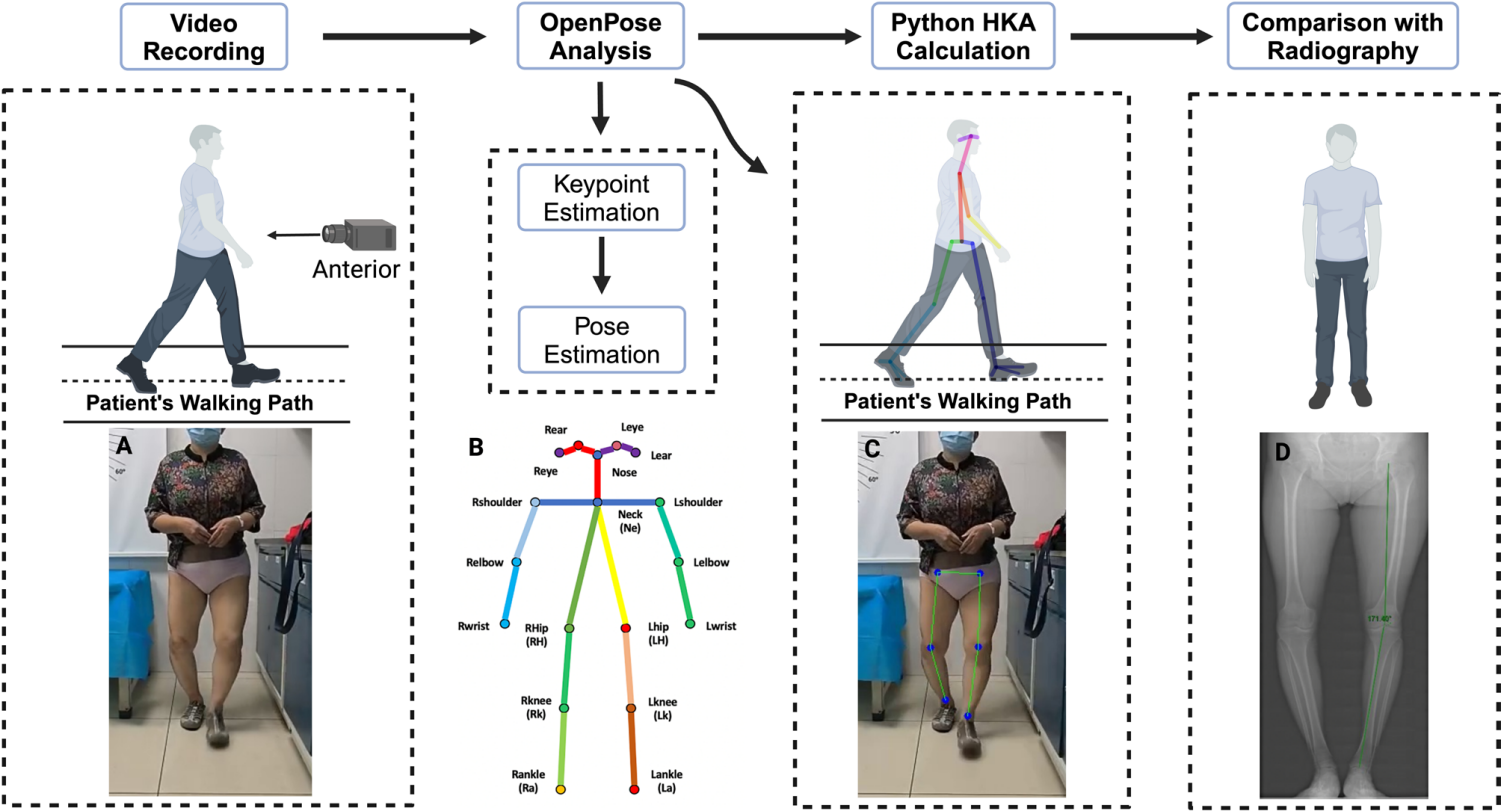
Workflow correspondent to the data processing using OpenPose. The illustration shows the workflow for video recording and OpenPose processing. First, a single smartphone captures the walking video of the subject. Then, OpenPose analyzes the video recording, and a custom Python script processes the data. Finally, compare the HKA angle with radiography. (**A**) Sample image from a participant’s recorded walk; (**B**) The OpenPose skeletal model and the name of each part. From: Sugiyama Y, Uno K, Matsui Y. Types of anomalies in two-dimensional video-based gait analysis in uncontrolled environments. PLoS Comput Biol. 2023;19(1):e1009989. (**C**) A sample output of OpenPose with the detected keypoints of interest plotted on the image; (D): Preoperative HKA angle measurement method in Radiographic image. The image was created using BioRender (https://biorender.com/) with publication and licensing rights (Agreement number: US27WFZL9I).

For the radiography measurements, knee radiography was recorded by radiology technicians unaware of the study’s objectives. On standard lower-limb weight-bearing full-length X-rays, the center of the femoral head, knee joint center, and ankle joint center were identified. The line connecting the femoral head center to the knee joint center was defined as the femoral mechanical axis, while the line connecting the ankle joint center to the knee joint center was defined as the tibial mechanical axis. HKA angle was formed by the mechanical axes of the femur and tibia (Fig. 1).

### Data analysis

We analyzed the test-retest reliability of two raters, A and B, using ICC (1,1). ICC values < 0.5, 0.5–0.75, 0.75–0.9, and > 0.9 indicate poor, moderate, good, and excellent agreement, respectively^10^. To evaluate validity, we compared pose estimation values with X-ray measurements. We used linear regression analysis to assess the potential of estimating radiographic values based on pose estimation values. R^2^ values of 0.01–0.09, 0.09–0.25, and > 0.25 indicate small, medium, and large correlations, respectively^11^. We then used ICC (2,1) and Bland-Altman analysis to assess the agreement between OpenPose and radiography^12^. All statistical analyses were conducted using SPSS software, with a significance level of *p* < 0.05.

## Results

We analyzed 50 knees (18 men and 32 women; mean age: 64.6 ± 5.2 years; mean HSS score: 54.03 ± 6.78).

The mean (standard deviation, SD) HKA angles measured by OpenPose and radiography imaging were − 6.76 (3.25) and − 6.89 (3.66), respectively. The mean (SD) of the difference between pose estimation and radiography is -0.131° (1.579). Intraclass correlation coefficient (ICC) for these two measurements are shown in Table 2. For test-retest reliability, the ICCs (1,1) of OpenPose and radiography were 1.000 and 0.983, respectively, both demonstrating good reliability.

**Table 2:** Test–retest reliability for openpose and radiography.

Measurement	Hip-Knee ankle (°)	ICCs(1,1)	95% CI for ICCs
OpenPose	− 6.76 ± 3.25	1.000	1.000–1.000
Radiography	− 6.89 ± 3.66	0.983	0.965–0.992

Values are presented as mean ± standard deviation.

*ICCs* intraclass correlation coefficients, *CI* confidence interval.

The correlation and consistency between the estimates and measurements are listed in Table 3. A large correlation was revealed in linear regression analysis (R^2^ = 0.814), and ICC (2,1) showed good agreement (0.897) between OpenPose and radiography.

**Table 3:** Regression models and intraclass correlation coefficients (2,1) between openpose and radiography, CI confidence interval, R^2^ coefficient of determination, ICCs intraclass correlation coefficients.

Measurement	Coefficients B	95% CI for B	*R* ^2^	ICCs (2, 1)	95% CI for ICCs
OpenPose × radiography	0.801	0.690–0.912	0.814	0.897	0.826–0.940

**Fig. 2:**
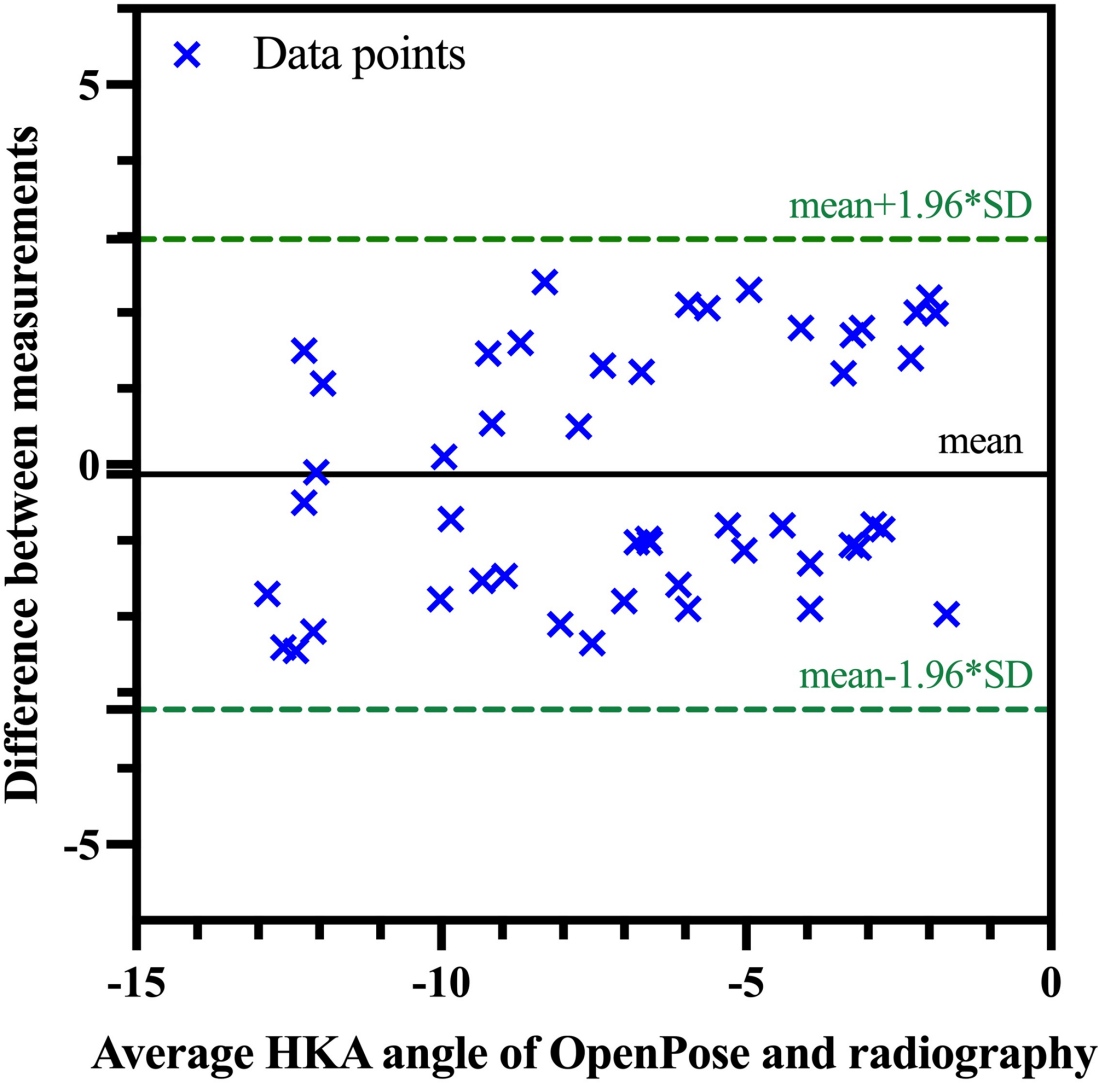
Bland–Altman plot for assessing measurement error. Mean hip-knee-ankle angle measurements for OpenPose and radiography are plotted on the x-axis and the difference between measurements on the y-axis. HKA, hip-knee-ankle.

Results of the Bland-Altman analysis are listed in Table 4; Fig. 2. The mean (SD) difference was − 0.131 (1.579) between OpenPose and radiography. The mean (SD) absolute difference was 1.441° (0.627) between OpenPose and radiography. The data points of all 50 patients fell within the limits of agreement, indicating consistency between the two methods. No significant difference was found between OpenPose and radiography (*p* = 0.560).

**Table 4:** Bland–Altman analysis between openpose and radiography.

Measurement	AD	MD	LOA (lower-upper)	95% CI for MD	*P*-values
OpenPose × radiography	1.441 ± 0.627	− 0.131 ± 1.579	6.190 (− 3.226 to 2.964)	− 0.580 to 0.318	0.560

Values are presented as mean ± standard deviation. AD mean of absolute difference, MD mean of difference, LOA 95% limits of agreement, CI confidence intervals.

## Discussion

This study demonstrates that OpenPose exhibits excellent test-retest reliability and strong agreement with radiographic measurements in assessing the HKA angle during dynamic walking. Compared to X-rays, OpenPose yielded a fixed error of 0.131° and an absolute error of 1.579°. We interpret these findings as supporting our hypothesis that this video-based, markerless method has potential as a reliable clinical alternative.

OpenPose functions by generating confidence maps for each joint across video frames using a deep learning model trained on a large-scale image dataset. It estimates joint positions by identifying peak values in these maps, allowing for consistent and reproducible measurements. Unlike radiography—the current gold standard for HKA evaluation—OpenPose offers a non-invasive, radiation-free, and easily accessible solution, particularly suitable for routine and remote gait assessments.

These results may be attributed, in our interpretation, to OpenPose’s robust pose-tracking algorithm, which benefits from deep learning architecture, particularly convolutional neural networks (CNNs), and specialized algorithmic design. CNNs extract low-level features (such as edges and textures) and high-level features (such as the shape and structure of human body parts) from images through multiple layers of convolution. Additionally, Part Affinity Fields (PAFs) are introduced to represent the relationships and connectivity between human keypoints. This approach not only accurately detects keypoints for a single individual but also maintains efficiency and precision in complex multi-person scenarios. However, this process represents a challenging problem, as the algorithm must remain robust to variations in scale, perspective, lighting, and even partial occlusion of body parts.

With regard to validity and reliability, OpenPose may yield better results than a 3D motion analysis system for HKA angle measurement. A study reported that a marker-based 3D motion analysis system had a random error of 2.4°and a fixed error of 3.6° in HKA angle measurement compared to full-length radiography, likely due to the estimation method for the hip center^13^. Additionally, the random error in this system is greater than that of OpenPose in this study, confirming OpenPose as a close approximation to the gold standard. Another study reported a high correlation (R^2^ = 0.90) when comparing HKA values measured by radiographs and the Opti-Knee 3D knee motion analysis system^14^while our study found R^2^ = 0.814. Though slightly lower than the 3D system, OpenPose is more user-friendly and does not require a lab setup, suggesting it could become an alternative to X-rays. Therefore, compared to traditional motion analysis equipment, OpenPose can detect the position and orientation of objects without the need for placing markers. This eliminates the requirement for a laboratory setup, making it more convenient and cost-effective than traditional portable devices, and better suited for clinical applications.

There remains considerable controversy regarding how to best restore individualized coronal alignment during TKA. To address this challenge, various classification systems have been proposed. For example, Lin et al. introduced 27 knee phenotypes^15^while Hirschmann et al. expanded this to 125 functional knee phenotypes that incorporate native lower limb alignment and individual anatomical variations^16^. In 2021, MacDessi et al. proposed the Coronal Plane Alignment of the Knee (CPAK) classification, which has further advanced the concept of personalized TKA^17^. However, all these systems are based on full-length, weight-bearing radiographs of both lower limbs, representing a static assessment method.

There is ongoing debate over whether the static HKA angle accurately reflects the coronal alignment of the knee throughout the dynamic gait cycle, especially during the key flexion peaks in mid-stance and mid-swing phases. Studies have shown that dynamic HKA tends to exhibit more pronounced varus or valgus deviations compared to static measurements, and these dynamic variations may significantly impact postoperative functional recovery, prosthesis longevity, and patient satisfaction. Therefore, the concept of dynamic HKA has been proposed to provide a more comprehensive and realistic evaluation of functional alignment, with important clinical implications.

In recent years, the rapid increase in personalized alignment classification systems reflects a shift from traditional static and standardized models to dynamic, individualized coronal alignment strategies. As Indelli pointed out, the proliferation of classification systems not only highlights the high complexity of knee joint kinematics but also reveals the limitations of static mechanical axis models in explaining functional alignment^18^. Our study focuses on the dynamic changes of the HKA angle throughout the gait cycle, aligning with the current emphasis on personalized and function-oriented TKA strategies, and further supports the necessity of incorporating dynamic alignment parameters in preoperative planning. Dynamic HKA, as a comprehensive indicator of lower limb motion status, holds potential to improve postoperative knee function, enhance patient satisfaction, and extend prosthesis lifespan.

We selected the HKA angle as the primary measurement parameter because it is the most commonly used and representative metric for assessing coronal alignment and is widely used to predict the risk of KOA progression. Research indicates that abnormal alignment (such as varus deformity) is closely associated with abnormal joint loading and cartilage degeneration. To achieve noninvasive measurement of dynamic HKA, this study is the first to apply OpenPose for HKA angle evaluation from dynamic gait videos. Unlike previous methods that extracted joint features from static images^19^we captured joint changes during walking, with a measurement reliability (ICC(2,1)) of 0.897, indicating good consistency. This method requires no specialized equipment or professional support, enabling patients to perform self-assessments at home. It holds promise as a convenient and practical tool for dynamic alignment evaluation, providing robust data support for personalized TKA strategies.

**Fig. 3:**
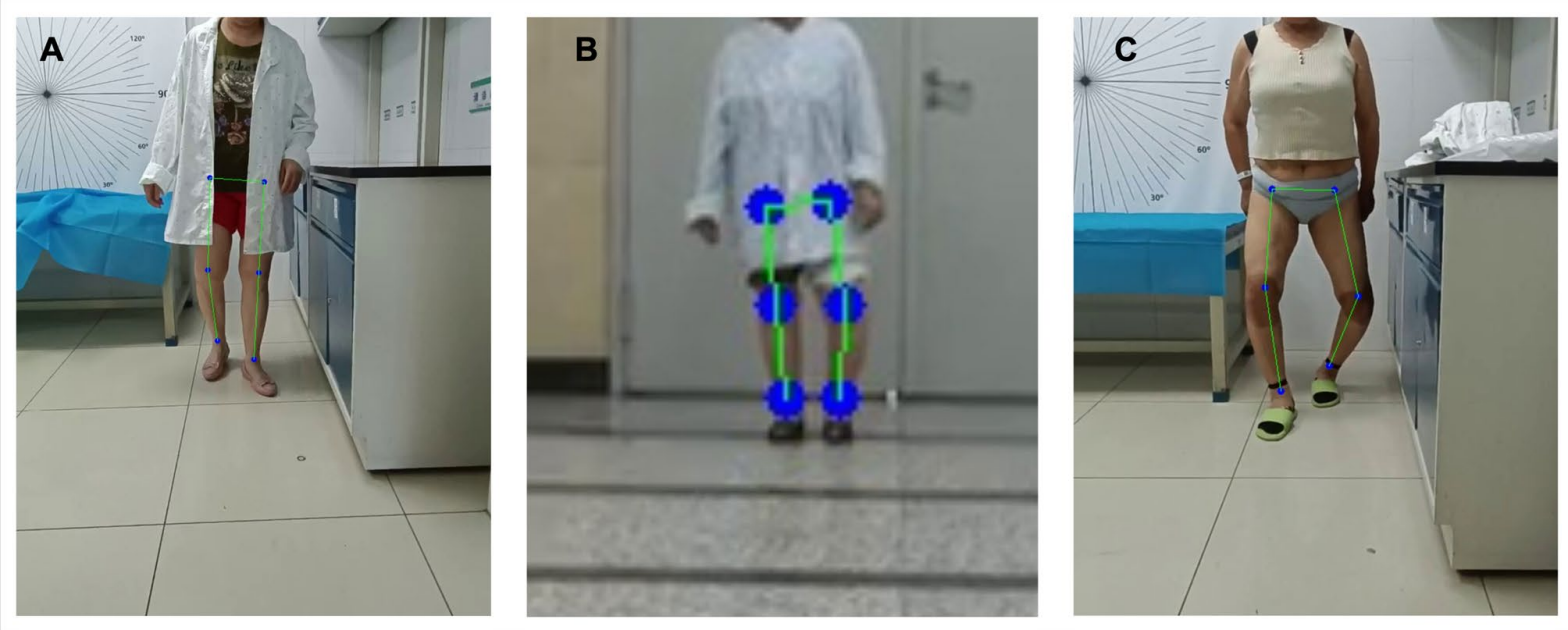
Examples of significant errors in OpenPose pose estimation. (**A**) Clothing and other factors, which are not accounted for in training data, significantly impact the accuracy of joint detection. (**B**) Large distances reduce OpenPose performance, leading to inaccurate joint localization and increased errors. (**C**) As the hip-knee-ankle (HKA) angle deviates further from 180 degrees, the accuracy of joint detection and HKA angle calculation decreases significantly.

This study has several limitations. First, OpenPose, originally developed for general-purpose pose estimation^20^, may be affected by clothing, occlusion, and excessive distance, reducing joint recognition accuracy (Fig. 3A,B). Additionally, as a 2D method, it lacks the depth needed for full 3D motion analysis, which limits its application in capturing complex joint dynamics during gait. Moreover, accuracy decreases when the HKA angle deviates significantly from neutral (180°), especially during dynamic walking. This may be due to the model’s training bias toward standard postures and increased joint occlusion in individuals with valgus or varus deformities (Fig. 3C). These factors should be considered when interpreting results for clinical use. Lastly, while OpenPose offers contactless and accessible skeletal tracking, future studies should explore its integration with tools such as sEMG or foot pressure systems to enhance its clinical utility and deepen our understanding of knee biomechanics.

What’s more, one methodological limitation of this study is that although normality was confirmed using the Kolmogorov–Smirnov test (*p* > 0.05), Levene’s test revealed significant differences in variance between raters (*p* < 0.05), indicating heteroscedasticity. We attempted to address this issue by applying common data transformation methods (e.g., logarithmic and square-root transformations), but the small sample size limited the stability and interpretability of the transformed results. Therefore, while we proceeded with the analyses, we advise interpreting the reliability estimates with caution. This limitation highlights the need for future studies to include larger sample sizes and consider alternative modeling strategies to better accommodate non-constant variances.

## Conclusions

Our study supports the hypothesis that for KOA patients, OpenPose is an effective tool to measure their HKA angle. OpenPose measurements match the precision of X-rays, suggesting its potential as a safer alternative or complementary tool for evaluating HKA angle changes and monitoring KOA progression. OpenPose provides a safe, cost-effective, and user-friendly solution for monitoring lower limb alignment, with promising applications in remote healthcare and rehabilitation management. In the context of future rehabilitation trends, OpenPose or advanced versions of this technology may contribute significantly to the expansion of rehabilitation services and the enhancement of health monitoring.

## Data Availability

The datasets used and/or analyzed during the current study are available from the corresponding author on reasonable request.
